# Post-neonatal Mortality, Morbidity, and Developmental Outcome after Ultrasound-Dated Preterm Birth in Rural Malawi: A Community-Based Cohort Study

**DOI:** 10.1371/journal.pmed.1001121

**Published:** 2011-11-08

**Authors:** Melissa Gladstone, Sarah White, George Kafulafula, James P. Neilson, Nynke van den Broek

**Affiliations:** 1 Department of Women's & Children's Health,Institute of Translational Medicine, University of Liverpool, Liverpool, United Kingdom; 2 Malawi-Liverpool-Wellcome Trust Clinical Research Programme, College of Medicine, University of Malawi, Malawi; 3 Department of Obstetrics & Gynaecology, College of Medicine, University of Malawi, Blantyre, Malawi; 4 Liverpool School of Tropical Medicine, Liverpool, United Kingdom; Cambridge University, United Kingdom

## Abstract

**Background:**

Preterm birth is considered to be associated with an estimated 27% of neonatal deaths, the majority in resource-poor countries where rates of prematurity are high. There is no information on medium term outcomes after accurately determined preterm birth in such settings.

**Methods and Findings:**

This community-based stratified cohort study conducted between May–December 2006 in Southern Malawi followed up 840 post-neonatal infants born to mothers who had received antenatal antibiotic prophylaxis/placebo in an attempt to reduce rates of preterm birth (APPLe trial ISRCTN84023116). Gestational age at delivery was based on ultrasound measurement of fetal bi-parietal diameter in early-mid pregnancy. 247 infants born before 37 wk gestation and 593 term infants were assessed at 12, 18, or 24 months. We assessed survival (death), morbidity (reported by carer, admissions, out-patient attendance), growth (weight and height), and development (Ten Question Questionnaire [TQQ] and Malawi Developmental Assessment Tool [MDAT]). Preterm infants were at significantly greater risk of death (hazard ratio 1.79, 95% CI 1.09–2.95). Surviving preterm infants were more likely to be underweight (weight-for-age z score; *p*<0.001) or wasted (weight-for-length z score; *p*<0.01) with no effect of gestational age at delivery. Preterm infants more often screened positively for disability on the Ten Question Questionnaire (*p* = 0.002). They also had higher rates of developmental delay on the MDAT at 18 months (*p* = 0.009), with gestational age at delivery (*p* = 0.01) increasing this likelihood. Morbidity—visits to a health centre (93%) and admissions to hospital (22%)—was similar for both groups.

**Conclusions:**

During the first 2 years of life, infants who are born preterm in resource poor countries, continue to be at a disadvantage in terms of mortality, growth, and development. In addition to interventions in the immediate neonatal period, a refocus on early childhood is needed to improve outcomes for infants born preterm in low-income settings.

*Please see later in the article for the Editors' Summary*

## Abstract 2

Using data collected as a follow-up to a randomized trial, Melissa Gladstone and colleagues show that during the first two years of life, infants born preterm in southern Malawi are disadvantaged in terms of mortality, growth, and development.

## Editors' Summary

**Background:**

Being born at term in Africa is not necessarily straightforward. In Malawi, 33 of every 1,000 infants born die in the first 28 days after birth; the lifetime risk for a mother dying during or shortly after pregnancy is one in 36. The comparable figures for the United Kingdom are three infants dying per 1,000 births and a lifetime risk of maternal death of one in 4,700. But for a baby, being born preterm is even more risky and the gap between low- and high-income countries widens still further. According to a World Health Organization report in 2010, a baby born at 32 weeks of gestation (weighing around 2,000 g) in Africa has little chance of survival, while the chances of survival for a baby born at 32 weeks in North America or Europe are similar to one born at term. There are very few data on the longer term outcomes of babies born preterm in Africa and there are multiple challenges involved in gathering such information. As prenatal ultrasound is not routinely available, gestational age is often uncertain. There may be little routine follow-up of preterm babies as is commonplace in high-income countries. Data are needed from recent years that take into account both improvements in perinatal care and adverse factors such as a rising number of infants becoming HIV positive around the time of birth.

**Why Was This Study Done?:**

We could improve outcomes for babies born preterm in sub-Saharan Africa if we understood more about what happens to them after birth. We cannot assume that the progress of these babies will be the same as those born preterm in a high-income country, as the latter group will have received different care, both before and after birth. If we can document the problems that these preterm babies face in a low-income setting, we can consider why they happen and what treatments can be realistically tested in this setting. It is also helpful to establish baseline data so that changes over time can be recorded.

The aim of this study was to document four specific outcomes up to the age of two years, on which there were few data previously from rural sub-Saharan Africa: how many babies survived, visits to a health center and admissions to the hospital, growth, and developmental delay.

**What Did the Researchers Do and Find?:**

The researchers examined a group of babies that had been born to mothers who had taken part in a randomized controlled trial of an antibiotic to prevent preterm birth. The trial had previously shown that the antibiotic (azithromycin) had no effect on how many babies were born preterm or on other measures of the infants' wellbeing, and so the researchers followed up babies from both arms of the trial to look at longer term outcomes. From the original group of 2,297 women who took part in the trial, they compared 247 infants born preterm against 593 term infants randomly chosen as controls, assessed at 12, 18, or 24 months. The majority of the preterm babies who survived past a month of age (all but ten) were born after 32 weeks of gestation. Compared to the babies born at term, the infants born preterm were nearly twice as likely to die subsequently in the next two years, were more likely to be underweight (a third were moderately underweight), and to have higher rates of developmental delay. The commonest causes of death were gastroenteritis, respiratory problems, and malaria. Visits to a health center and admissions to hospital were similar in both groups.

**What Do these Findings Mean?:**

This study documents longer term outcomes of babies born preterm in sub-Saharan Africa in detail for the first time. The strengths of the study include prenatal ultrasound dating and correct adjustment of follow-up age (which takes into account being born before term). Because the researchers defined morbidity using routine health center attendances and self-report of illnesses by parents, this outcome does not seem to have been as useful as the others in differentiating between the preterm and term babies. Better means of measuring morbidity are needed in this setting.

In the developed world, there is considerable investment being made to improve care during pregnancy and in the neonatal period. This investment in care may help by predicting which mothers are more likely to give birth early and preventing preterm birth through drug or other treatments. It is to be hoped that some of the benefit will be transferable to low-income countries. A baby born at 26 weeks' gestation and admitted to a neonatal unit in the United Kingdom has a 67% chance of survival; preterm babies born in sub-Saharan Africa face a starkly contrasting future.

**Additional Information:**

Please access these Web sites via the online version of this summary at http://dx.doi.org/10.1371/journal.pmed.1001121.

- UNICEF presents useful statistics on mother and child outcomes
- The World Health Organization has attempted to analyse preterm birth rates worldwide, including mapping the regional distribution and has also produced practical guides on strategies such as Kangaroo Mother Care, which can be used for the care of preterm infants in low resource settings
- Healthy Newborn Network has good information on initiatives taking place to improve neonatal outcomes in low income settings
- The March of Dimes, a nonprofit organization for pregnancy and baby health, provides information on research being conducted into preterm birth
- Tommy's is a nonprofit organization that funds research and provides information on the risks and causes of premature birth

## Introduction

An estimated 3.6 million neonatal deaths occur each year, accounting for 41% of deaths in children under 5 y old [1]. Over three-quarters of these deaths occur in South Asia and sub-Saharan Africa, with causes of death often poorly documented. Prematurity (birth at less than 37 completed wk gestation) is considered to be associated with an estimated 27% of all neonatal deaths [2], but estimates are difficult because of uncertainties about gestational age in many low-income countries. The very few studies using accurate, prenatal ultrasound dating in Sub-Saharan Africa, including our own, have reported a high incidence (15%–22%) of preterm births [3]–[5].

There is good evidence that antenatal corticosteroids and improved care in the immediate neonatal period, such as Kangaroo Mother Care, and encouragement of early breastfeeding improve neonatal survival in the first month of life [6],[7]. There are recent estimates of post-neonatal mortality worldwide [8]; however, the information specifically about mortality and morbidity of the surviving preterm infant in these settings is largely unavailable.

Most previous studies come from high-resource countries and have focused on babies born very preterm, before 32 weeks' gestation. Almost three-quarters of preterm births occur however between 32 and 36 wk [9],[10], and these late preterm infants are still at greater risk of infant mortality and morbidity compared to infants born at term [11]. A comprehensive search of the literature has identified few studies reporting medium or longer term outcomes for babies born preterm in low-income settings. All these studies have used proxy measures for gestational age (low birth weight, Dubowitz scoring, or other) [12]–[15] with many of the studies only reporting hospital-based births. None used prenatal ultrasound—the most accurate method of assessing gestational age.

The aim of our study was to assess four specific outcomes—post-neonatal survival, morbidity, growth, and development—in a community-based sample of infants born after spontaneous preterm delivery in rural sub-Saharan Africa. All had gestational age accurately determined using prenatal ultrasound scanning.

## Methods

### Ethics Statement

We obtained written permission from each district health officer for the four areas in which the study was conducted. The research midwives explained the purpose of the study to each child's parent or carer and obtained written informed consent to participate in the study. The study gained ethical approval from the College of Medicine Research Ethics Committee in Malawi.

### Malawi in Context

Malawi is considered to be one of the poorest countries in the world with a per capita income of US$290 per year [16]. 80% of the population live in rural areas and livelihoods are earned mainly through subsistence farming. The estimated neonatal mortality rate is 33/1,000 live births with just over 80% of births delivered in a health facility [17]. More than 95% of women attend for antenatal care in the community, mostly in community-based health centres [18]. Antenatal steroids are not routinely given to women in Malawi prior to preterm birth. Malawi has very limited neonatal care with few units providing special care for preterm infants. Those units that exist have oxygen, antibiotics, and incubators and very little in the way of diagnostic facilities. In recent surveys, 72% of children under 6 mo were exclusively breastfed and by 6–9 mo, 87% of infants are given complementary foods [17].

### Study Setting and Population

We carried out a cohort study assessing mortality, morbidity, development, and growth in post-neonatal infants who were known to have been born preterm (<37 completed wk gestation by ultrasound scan) (group 1) or at term (37–41 wk) (group 2). All infants for this follow-up study were born during a placebo-controlled double blind antibiotic intervention study (APPLe trial ISRCTN84023116) in southern Malawi. Birth weights were recorded for those who delivered in a health facility (65.5%). In the parent cohort population, low birth weight (<2.5 kg) was recorded in 10.0% of babies. Serial ultrasound was not available for this population, therefore making it impossible to diagnose infants as being small for gestational age (due to intrauterine growth restriction or other) during pregnancy. Nutritional status in women was measured using BMI (kg/m^2^) with a mean (standard deviation [SD]) BMI of 22.7 (2.7). More details of the APPLe cohort can be found elsewhere [4].

**Figure 1:**
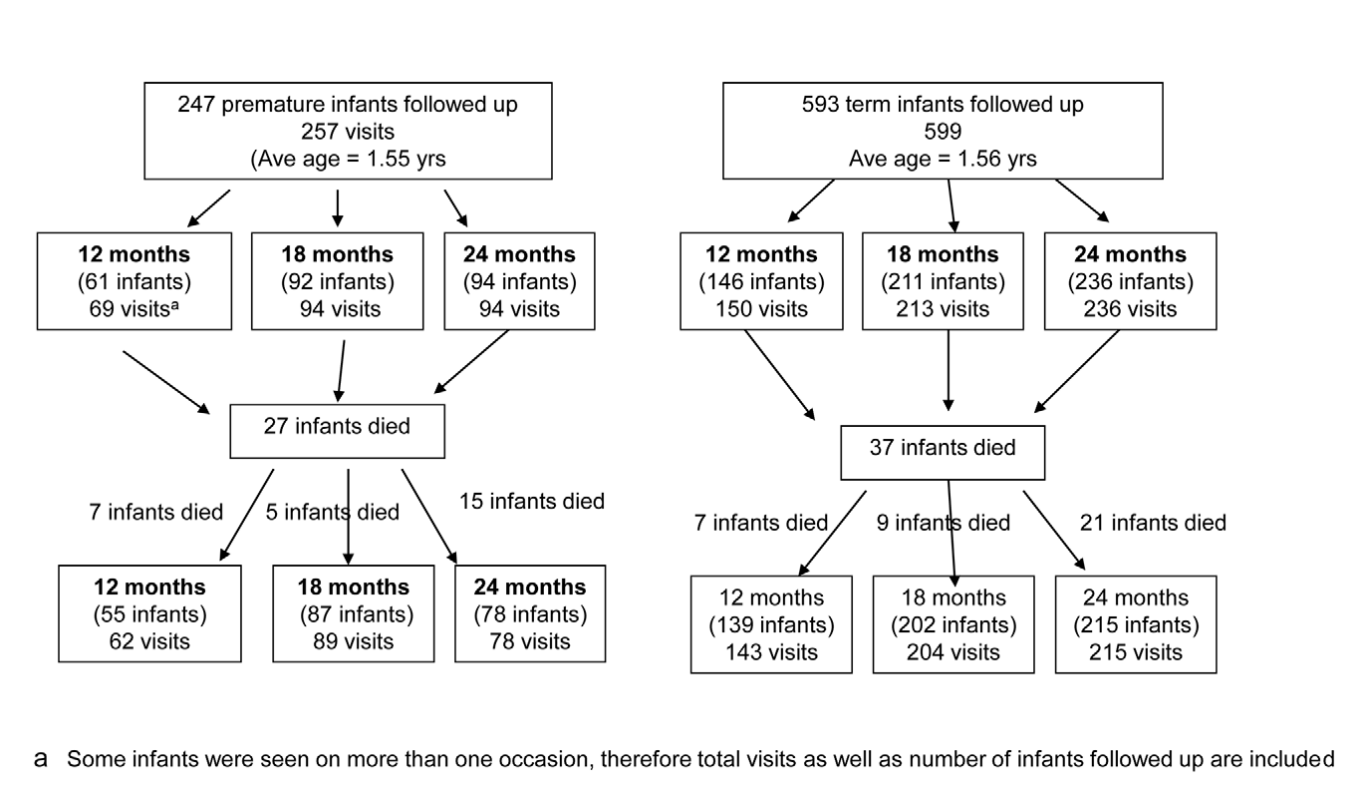
Flow chart of post-neonatal infants followed up in community cohort study.

Two strata were defined: babies born preterm and babies born at term. We attempted to follow up babies who were known to have survived the first 6 wk of life. The entire set of post-neonatal surviving preterm babies born preterm from the APPLe trial cohort of women was included. There were 295 preterm babies identified for follow-up including 27 twin babies (14 pairs, one died). 48 (16.3%) infants known to have been born preterm and to have survived the neonatal period were lost to follow-up (moved from area, could be not traced). For the comparison cohort, we selected at least double the number of term-born post-neonatal babies. This selection was done using a computer-generated random list from the APPLe parent cohort. Following this method, 678 babies born at term were identified. 593 (87.5%) were found at follow-up and included in the study. 85 infants known to be born at term (12.6%) were lost to follow-up (moved from the area, could be not traced) (Figure 1).

We aimed to assess all infants on at least one occasion at a corrected age of 12 or 18 or 24 mo. We assessed them at this precise age ±1 wk. Corrected age was defined as chronological age (time elapsed after birth) minus number of weeks born less than 40 wk [19]. All assessments were carried out over a period of 7 mo from May 2006 until December 2006 using a team of six research midwives. 16 infants (ten preterm and six term) were assessed on two occasions 6 mo apart. These assessments are included in the analyses of development. We present the analyses below, with and without twins included.

### Data Collection

Socio-demographic characteristics were gathered for each family by interviewing the mother at the time of assessment, using a standard set of questions similar to those used in the Malawi Demographic Health Survey [20] as recommended by the World Bank [21].

To assess survival, we collected information on date of death, cause of death, and place of death by interviewing the mother. Cause of death was established using information gathered at verbal autopsy and classified as recommended in recent studies [22].

To assess morbidity, we collected data using a structured questionnaire. This questionnaire included whether the child was well at the time seen (as reported by the mother and observed by the research nurse midwife), the number of episodes and reasons for accessing care at a health facility, the number and reasons for being admitted to hospital in the past, and, specifically, whether the child had ever had convulsions.

Growth was assessed for each child by measuring length and nude weight. Weight was measured using electronic infant weighing scales (SECA 735) with reading increments of 10 g. Length was measured to the nearest 0.5 cm using standard locally made length measurement boards based on blueprints for their construction from the World Health Organization and the US Centers for Disease Control (Atlanta, Georgia) [23]. All growth data were collected by the research nurse midwives who had been specifically trained prior to the start of the study. Training used the “my measure” formulas for anthropometeric standardisation as recommended by the Anthropometric Indicators Measurement Guide [24]. At recommended stages of training, ten children had their height and weight measurements repeated on two occasions by each research midwife. Training continued with the research midwives until satisfactory accuracy was obtained. We asked each mother whether the child was still breastfeeding at the time of assessment.

Development and disability were assessed using the Ten Question Questionnaire (TQQ) [25] and the Malawi Developmental Assessment Tool (MDAT), which we have described previously [26]. The MDAT has demonstrated good reliability, construct validity, and sensitivity in predicting moderate to severe neurodisability and developmental delay in a Malawian population of malnourished children. We used the MDAT to assess children in two ways: through a pass/fail scoring system and through a numerical scoring system applied to each of four domains of development [26]. Only eight of the ten questions in the TQQ were used as the children were all under 2 y and questions 8 and 9 regarding speech and language were not appropriate for this age group. We were unable to specifically assess for cerebral palsy, as this is a clinical diagnosis that without further assessments and imaging was not able to be diagnosed in this rural African setting.

### Statistical Analysis

All data were double entered and verified, hosted on a password-protected SQL server with any discrepancies and outlying results reviewed. Mothers and babies were identified only by the mother's identification number and in the case of twins, the twin number. Data were analysed using SPSS for Windows version 17 and Stata version 10. All available data were included in the analyses.

Analyses compared data for infants and their mothers in the preterm and term delivery groups. All infants were used in the analysis, except where stated otherwise. Survival of infants in each of these strata was estimated using Kaplan-Meier curves and compared using a Cox's proportional hazards model. Gender was also included in the Cox's proportional hazards analysis. When date of death was not reported (six cases) it was assumed to have been mid-way between birth and the assessment visit. For all other analyses, when data required for an analysis was missing the record was omitted from analysis. Socio-economic status was measured using principal components analysis of multiple assets following methods from the World Bank [21],[27]. Socio-economic quintiles for the two groups of mothers were analysed using logistic regression with term/preterm as the binary outcome.

Weight-for-corrected-age (WAZ), height-for-corrected-age (HAZ), and weight-for-height z (WHZ) scores were derived using Epi-info version 3.2.2 with World Health Organization reference data [28],[29]. All analyses, except survival analyses, used corrected age (defined above). Growth data and MDAT scores were each analysed using linear regression models with gestational age at delivery as a covariate in the model for both sets of scores; age at assessment was an additional covariate in the analysis of MDAT scores. Stratified Mantel-Haenszel tests were used to examine the association of each of three indices of growth (WAZ and length-for-age [LAZ] and weight-for-length [WLZ] z scores), and the number of times health care was accessed with prematurity, stratified by age of assessment. Other binary or categorical outcomes were analysed using Pearson chi square tests unless otherwise stated.

Stratified Mantel-Haenszel tests were also used to examine the association between being underweight (WAZ<−2) and identification of disability on the TQQ or severe developmental delay on the MDAT. Stratification was by whether preterm or not and by age of assessment to control for the influence of these factors.

## Results

### Population Followed Up

**Figure 2:**
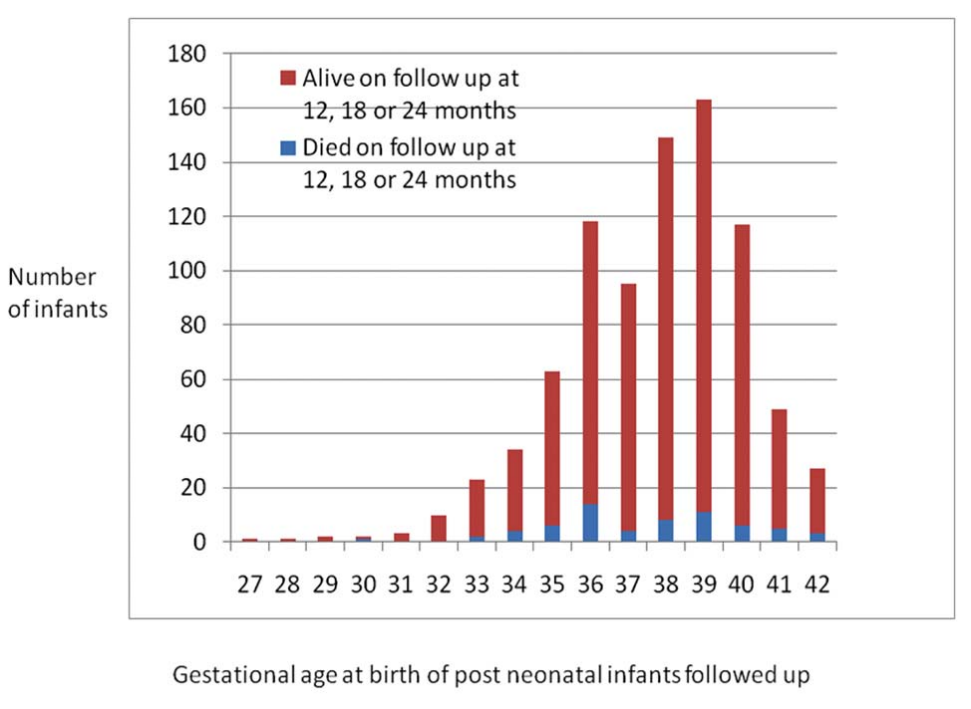
Number of post-neonatal infants followed up by gestational age at birth.

A total of 840 post-neonatal infants were followed up, of whom 247 were born preterm and 593 were born at term (Figure 1). Among these, 12 pairs of twins were born preterm (24 babies) and one set of twins (two babies) were born at term. Among preterm births, gestational age at birth (given in completed weeks of gestation) was above 34 wk for 203/247 infants (82.2%), either 32 or 33 wk for 34/247 infants (13.8%), and between 28 to 31 wk for 9/247 (3.6%) with only one infant born below 28 wk. Figure 2 shows the distribution of gestational age at birth of the cohort followed up. At time of assessment, the mean corrected ages for the two groups were equal. Female babies were more prevalent in the preterm group (58%) than in the term group (50%).

### Survival

**Figure 3:**
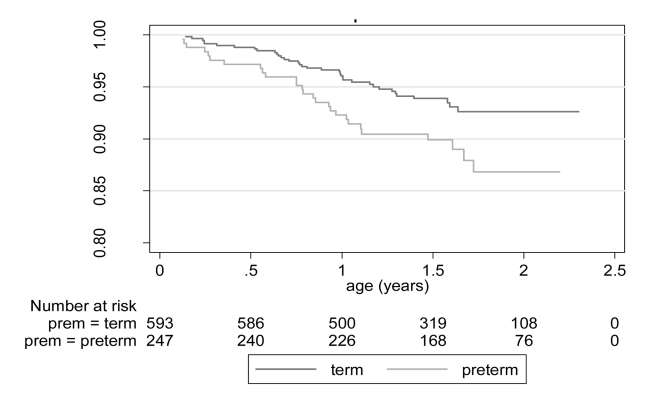
Kaplan Meyer curves: survival of post-neonatal infants born preterm and at term (likelihood ratio X^2^ statistic: 5.05; *p* = 0.02).

Post-neonatal infants born preterm were at significantly greater risk of death (during the 6-wk to 24-mo follow-up period) than those born at term: 27/247 (10.9%) preterm and 37/593 (6.2%) term-born infants died; Cox PH χ^2^ = 5.05, *p* = 0.02. The estimated hazard ratio (95% CI) for preterm compared with term-born infants is 1.79 (1.09–2.95) (Figure 3). With twin preterm-born infants excluded, Cox PH χ^2^ = 2.76, *p* = 0.10 and hazard ratio is 1.58 (0.93–2.69). The estimated mortality rates for infants born preterm who had survived the neonatal period was almost double that of infants born at term. At 1 y, there was an estimated mortality rate of 7.7% (5.0–11.8) for preterm and 4.0% (2.7–6.0) for term infants, and at 2 y (cumulative) a rate of 13.2% (8.9–19.3%) and 7.6% (5.4–10.6), respectively (Table 1). Mortality rates were also significantly higher for boys compared with girls. Cox PH χ^2^ = 9.23, *p* = 0.002; the estimated hazard ratio (95% CI) for male compared with female infants is 2.19 (1.30–3.68).

**Table 1:** Mortality rates at 1 and 2 y for infants born preterm or at term (by gender) with twins included in analysis.

Age	Grouping	Preterm	Term
**1 y**	Overall	7.7% (5.0%–11.8%)	4.0% (2.7%–6.0%)
	By gender		
	Boys	8.8% (4.7%–16.2%)	5.5% (3.4%–8.8%)
	Girls	6.9% (3.8%–12.5%)	2.5% (1.0%–5.2%)
**2 y**	Overall	13.2% (8.9%–19.3%)^a^	7.6% (5.4%–10.6%)
	By gender		
	Boys	16.8% (9.9%–27.9%)	11.2% (7.7%–16.3%)
	Girls	10.5% (5.9%–18.3%)	3.8% (1.9%–7.6%)

a Without twins: 12% (7.8%–18.3%).

There were very few babies born with a gestational age under 32 wk (very preterm) who survived the post-neonatal period in our cohort (*n* = 10). Within this group, only one of these was found dead on follow-up (Figure 2). The numbers are small, but when analysed, we found no significant differences in mortality between babies born less than 32 wk and those born between 32 and 37 wk (Pearsons chi 0.439).

Information on cause of death was available for 23 of 27 (85.2%) infants born preterm and 37 of 37 (100%) infants born at term (Table 2). We identified five main causes of death in post-neonatal infants: gastroenteritis (36.7%), malaria (15%), respiratory problems (15%), anaemia (15%), and fever (6.7%). In the preterm group there were more reported deaths due to respiratory problems (17.4% versus 13.5) and fewer due to gastroenteritis (26.1% versus 43.2%) but the differences were not statistically significant. By gender, more term males than females died of gastroenteritis, malaria, and fever. In the preterm group, more females died of respiratory illness (four versus 0), but more males died of other conditions such as malaria and fever. None of these differences were statistically significant.

**Table 2:** Cause of death by verbal autopsy of babies born at term and preterm (by gender).

Cause of Death^a^	Preterm		Term	
	Boys	Girls	Boys	Girls
Total *n* infants who died	14	13	28	9
Gastroenteritis (diarrhoea and vomiting)	3	3	11	5
Respiratory problem	0	4	3	2
Anaemia	1	1	1	1
Malaria	2	2	5	0
Fever	2	0	2	0
Other	4	1	6	1
Total *n* causes of death reported (by gender)	12	11	28	9

a Total *n* causes of death reported: preterm, 23; term, 37.

### Morbidity

Mothers did not commonly report morbidity at the time of assessment (12, 18, or 24 mo) with 98.7% (226/229) of the preterm group and 97.9% (550/562) of the term group reported to be well at the time of interview. Despite this, reported attendance to the health centre was high in both infants born preterm (94.3%) and those born at term (91.3%) with no significant difference between the groups. The (median) numbers of times that health care was accessed were 3, 3, and 4 times at 12, 18, and 24 mo of age, and did not differ significantly between the groups (Mantel-Haenszel *M* ^2^ = 0.03, *p* = 0.86). Nonroutine reasons for attendance to the health centre included malaria, respiratory problems, diarrhoea, upper respiratory tract infections, skin rashes, and fever. Only two mothers reported going to the health centre because their child was malnourished. Visits for routine immunisations (97.4% [preterm] versus 98.9% [term]) and weighing 82.5% (preterm) versus 85.6% (term) were found to be similar.

There were no significant differences in the preterm versus term group in the number of infants reported to have been admitted to a hospital (58/230; 25.2% versus 116/559; 20.8%). The median number of admissions was 1.0 in each of the groups. Infants born preterm were more likely to be admitted for cerebral malaria (3.5% versus 1.8%) or anaemia (3.9% versus 2.1%), but this was not statistically significant. Similar proportions were admitted for simple malaria (9.2% versus 9.9%) and pneumonia (5.7% versus 5.0%). Other causes for admission were sepsis (reported only in four cases), meningitis (one case), and malnutrition (five cases). HIV/AIDS was not reported by parents as a cause of admission to hospital. There were no significant differences in the number of children experiencing convulsions, as reported by parents, preterm compared to term-born children: 10.4% (22/211) versus 6.9% (38/548) (*p* = 0.13).

### Growth

When assessing growth/nutritional parameters for the cohorts, preterm infants were significantly more likely to be underweight (*p*<0.001). Almost one-third (32.3%) of preterm infants were moderately underweight (below −2 SD WAZ) and 11.1% severely underweight (below −3 SD WAZ) (Table 3). Preterm infants were also more likely to be moderately wasted (WLZ below −2 SD), with a quarter (24.9%) of preterm infants moderately wasted although the groups had similar levels of severe wasting (WLZ>3 SD below mean). In assessing for the effect of gestational age on the growth parameters, the estimated increase in WAZ and WLZ scores per additional week of gestational age was 0.08 (0.05–0.11) and 0.07 (0.02–0.11), respectively. Stunting (below −2 SD for LAZ) was common in both term- (38.3%) and preterm- (42.6%) born children; the effect of each additional week of gestational age at delivery (0.05 [−0.005 to 0.10]) was not significant (*p* = 0.08).

**Table 3:** Summary of WAZ, LAZ, and WLZ for preterm and term babies with estimated effects of gestational age at delivery from linear regression.

Growth Measurement	Category	Proportions Undernourished			Regression Analysis	
		12–23 mo Children MDHS	Preterm	Term	Effect of Gestational Age (wk) (95% CI)	*p*-Value
**WAZ**	**<**−**2 SD**	28.8%	73/226 (32.3%)	122/556 (22.9%)	0.078 (0.045–0.111)	<0.001
	**<**−**3 SD**	7.4%	25/226 (11.1%)	25/556 (4.5%)	—	—
**WLZ**	**<**−**2 SD**	6.8%	56/225 (24.9%	91/551 (16.5%)	0.065 (0.016–0.114)	0.01
	**<**−**3 SD**	1.8%	16/225 (5.9%)	33/551 (6.0%)	—	—
**LAZ**	**<**−**2 SD**	60.7%	98/230 (42.6%)	213/556 (38.3%)	0.046 (−0.005 to 0.097)	0.08
	**<**−**3 SD**	30.3%	45/230 (19.6%)	79/556 (14.2%)	—	—

MDHS, Malawi Demographic Health Survey [20].

Breastfeeding rates for preterm infants (100%) and term infants (98.6%) at 12 mo were very similar (*p* = 1.0). There was a trend for lower rates of breastfeeding in the preterm infants at 18 mo (80.7%) versus term infants (89.6%) (*p* = 0.057) and also at 24 mo for preterm (44.2%) versus term infants (48.3%) (*p* = 0.59), but neither were significant.

### Development

The post-neonatal infants born preterm were significantly more likely than term-born infants to screen positive overall on the TQQ (Table 4) (*p* = 0.002), with 32/230 (13.9%) of the preterm infants and 38/562 (6.8%) of the term infants scoring positive on one or more questions. Serious delay in walking, standing, or sitting (question 1) (*p* = 0.049) or being slow in comparison to children of the same age (neurodevelopmentally delayed; question 10) (*p* = 0.005) were the items that were significantly more frequent in infants.

**Table 4:** Number and percentage of children positive on the TQQ for each question and overall scored positive in one or more question.

Question Number	Reason for Being Positive on TQQ	*n* Preterm (*n* = 230) (%)	*n* Term (*n* = 562) (%)	Total *n* (*n* = 792) (%)	*p*-Value
1	Serious delay in sitting, standing, or walking	20 (8.7)	28 (5)	48 (6)	0.049
2	Difficulty seeing (daytime or night)	1 (0.4)	5 (0.9)	6 (1)	0.34
3	Difficulty hearing	1 (0.4)	1 (0.2)	2 (0.2)	0.516
4	Problems understanding what you are saying	3 (1.5)	2 (0.4)	5 (0.6)	0.121
5	Difficulty walking or moving his/her arms or weakness or stiffness in arms and legs	5 (2.2)	7 (1.3)	12 (1.5)	0.332
6	Child sometimes has fits, become rigid, or lose consciousness	9 (3.9)	11 (2.0)	20 (2.5)	0.112
7	Child not learning to do things like others at his age?	9 (3.9)	13 (2.3)	22 (2.8)	0.221
10	Does the child appear mentally backward or slow compared to others of same age?	11 (4.8)	8 (1.4)	19 (2.4)	0.005
Scored positive in one or more of above questions	—	32 (13.9)	38 (6.8)	70 (8.8)	0.002

In terms of overall pass/fail on the MDAT, more children in the preterm group compared to the term group failed the MDAT at each stage of assessment; at 12 mo this was 6.7% versus 2.9% (*p* = 0.216), at 18 mo 22.8% versus 10.9% (*p* = 0.009), and at 24 mo 12.8% versus 10.7% (*p* = 0.274). Significant differences were also found specifically at 18 mo for language development (*p* = 0.033) (Table 5). When development was assessed on the MDAT using numerical scores, there were significant changes with gestational age (wk) at delivery in gross motor (0.15/wk; *p* = 0.02), social development (0.24/wk; *p* = 0.004), and overall development at 18 mo (0.79/wk; *p* = 0.01), and in social development (0.21/wk; *p* = 0.02) at 24 mo (Table 6).

**Table 5:** Comparisons of mean MDAT scores and percentage passing/failing for each domain of development, for preterm and term babies, by age at assessment.

Domain of Development	Statistic	Age of Assessment (mo)								
		12 mo			18 mo			24 mo		
		Preterm *n* = 62^a^	Term *n* = 143^b^	*p*-Value	Preterm *n* = 89^c^	Term *n* = 205^d^	*p*-Value	Preterm *n* = 79^e^	Term *n* = 215^f^	*p*-Value
Gross motor	Percent fail	4.8%	3.5%	0.649	12.5%	8.3%	0.261	3.8%	3.3%	0.806
	Score	14.4	14.7	0.433	18.5	19.2	0.023	19.9	20.3	0.201
Fine motor	Percent fail	1.7%	0.7%	0.535	3.5%	1.6%	0.300	9.1%	3.4%	0.053
	Score	16.1	15.8	0.558	18.7	19.0	0.443	20.2	21.1	0.062
Language	Percent fail	1.6%	0.7%	0.552	2.2%	0%	0.033	2.6%	1.5%	0.554
	Score	10.2	10.4	0.551	12.6	12.5	0.909	14.9	15.9	0.017
Social	Percent fail	0%	0%	—	6.8%	5.5%	0.655	3.8%	5.7%	0.513
	Score	12.6	12.7	0.721	16.7	17.8	0.011	20.4	21.3	0.04
Total	Percent fail	6.7%	2.9%	0.216	22.8%	10.9%	0.009	12.8%	10.7%	0.274
	Score	53.1	52.9	0.93	66.3	68.3	0.04	75.5	77.7	0.081

a For each domain up to two (3.3%) children were not assessed.

b For each domain up to six (4%) children were not assessed.

c For each domain up to four (4.5%) children were not assessed.

d For each domain up to 13 (6.3%) children were not assessed.

e For each domain up to two (2.5%) children were not assessed.

f For each domain up to 19 (8.8%) children were not assessed.

**Table 6:** Summary of linear regression estimates of effect of gestational age at delivery and assessment age for MDAT scores.

Domain of Development	*n 12 mo, 18 mo, 24 mo*	Estimate of Increase in MDAT Score per Additional Week of Gestational Age					
		12 mo		18 mo		24 mo	
		*p*-Value	Effect (95% CI)	*p*-Value	Effect (95% CI)	*p*-Value	Effect (95% CI)
Gross motor	205, 293, 294	0.74	−0.02 (−0.15 to 0.10)	0.02	0.15 (0.03–0.27)	0.08	0.10 (−0.01 to 0.21)
Fine motor	200, 281, 280	0.12	−0.15 (−0.35 to 0.04)	0.57	0.05 (−0.12 to 0.21)	0.20	0.11 (−0.06 to 0.28)
Language	202, 289, 273	0.77	0.01 (−0.07 to 0.10)	0.69	0.02 (−0.07 to 0.11)	0.06	0.14 (−0.01 to 0.28)
Social	204, 289, 289	0.89	−0.01 (−0.17 to 0.15)	0.004	0.24 (0.08–0.41)	0.02	0.21 (0.04–0.38)
Overall	205, 294, 295	0.42	−0.17 (−0.59 to 0.24)	0.01	0.79 (0.10–0.87)	0.09	0.41 (−0.06 to 0.88)

Age at assessment was included as a covariate in these analyses.

The number of children demonstrating severe delay on the MDAT (delay greater than 2 SD from the mean expected score for the age of the child) was the same across the two groups (9/230 preterm infants [3.9%] versus 23/562 term infants [4.1%]).

### Growth and Development

We analysed the association between growth and development in this cohort. We used WAZ as these were the most robust of our three measures. There was a significant association between being underweight and positive on the disability screen (overall odds ratio [OR], 95% CI 2.88 [1.81–4.61]), or having severe delay on the MDAT (<−2 SD) (overall OR, 95% CI 4.06 [1.98–8.32]). The association was similar for both term and preterm infants (Table 7).

**Table 7:** Associations between being underweight (WAZ<−2) and developmental delay assessed by the TQQ and the MDAT for all babies and by preterm or not status.

Assessment	Cohort	OR^a^	95% CI	*p*-Value
**Positive score on TQQ**	All	3.18	1.92–5.29	<0.001
	Term babies	3.80	1.93–7.45	<0.0001
	Preterm	2.59	1.19–5.63	0.013
**MDAT score <−2 SD from the normal range.**	All	4.06	1.98–8.32	<0.0001
	Term babies	4.95	2.09–11.7	<0.001
	Preterm	2.63	0.69–10.1	0.14

a Stratified by age at assessment and, for all babies by whether premature or not.

## Discussion

This study has clearly shown that infants who are born prematurely in a rural community setting in sub-Saharan Africa, who survive the first month of life, are still up to twice as likely to die as term infants during the first 2 y of life with a cumulative mortality rate of 132/1,000 in comparison with those born at term (76/1,000). This cohort of preterm infants is unique because gestational age was established by ultrasound dating. We have shown that these infants are almost entirely a population of late preterm infants, but despite this, are still more likely to have higher rates of malnutrition and developmental delay than term infants. As rates of prematurity are high in sub-Saharan African settings [3]–[5],[30], this study provides evidence to show how crucial it is to concentrate on improving outcomes within this group.

Recent studies from high-income countries have also demonstrated this relatively increased mortality in late preterm babies; however, outcomes have mainly been measured only in the neonatal period [11]. Studies have suggested that causative factors may include thermal instability, hypoglycaemia, respiratory distress, apnoea, jaundice, and feeding difficulties [31]. There is a dearth of evidence from developing countries, but it could be surmised that infants born in the late preterm period in rural African settings are likely to be at increased risk for many of these problems with high levels of poverty, poor post-natal care, and high infection rates. Moreover, the population of infants in our study were not exposed to antenatal steroids and will be more likely to suffer from respiratory distress syndrome and related respiratory disorders [32]. These late preterm infants may also be malnourished due to feeding difficulties that cannot be augmented through supplementary feeding methods present in high-income settings. Poor nutrition will affect immune status and further lead to an increased risk of infection. There is some limited evidence that extra care of preterm-born infants in Asia, such as home care, skin-to-skin contact, and additional support for breastfeeding, may have some potential to prevent death in this group [7],[33],[34]. At the time of this study, Kangaroo Mother Care was limited in the community and district health care settings in Malawi, but services are now being scaled up and hopefully may improve rates of post-neonatal mortality in these late preterm-born infants [35].

Male infants within our cohort of infants born at term were more likely to die than female infants in the post-neonatal period. There is limited evidence that gender does have an effect on perinatal, neonatal [36], and under five death rates [37], but the reasons for this are not clear.

There is little information on cause of death in infants in resource-poor settings, particularly in early infancy. Verbal autopsy tools in this population were found to be useful and we documented more deaths in preterm infants associated with respiratory problems compared to infants born at term. In the absence of an agreed definition and framework for measuring morbidity in community settings in children, we used reported health-seeking behaviour as a proxy measure. Almost all mothers reported their children as well at the time of the study taking place. Almost all mothers had visited a health centre for routine weighing and immunisation and almost one in four children had been admitted as an inpatient in the first 2 y of life. There was no difference in health-seeking behaviour between babies born preterm and those born at term. The reports from the mothers of their children being “well” at the time of the study was surprising considering the morbidity we then encountered in terms of malnutrition, developmental delay, and the high rates of admission to the health centre. Our tools for assessing morbidity were clearly not specific enough and highlight that parent-reported morbidity is not a good measure of childhood morbidity. Currently, an agreed framework or standard for measuring morbidity in children is not available and further studies using other approaches are very much warranted [38].

In our cohort, children born preterm were significantly more likely to be underweight and wasted and did not demonstrate catch-up growth in contrast to those born at term. Comparison with studies from high-income settings is difficult as most have studied very preterm or low birth weight infants [39],[40]. We have identified one other community cohort study from a middle-income country (Brazil) reporting a 3% rate for being underweight with a comparison to terms of 0.8% [41]. Rates in our study were much higher (32.3% for term infants and 22.9% for preterms), suggesting a much higher overall level of malnutrition in this rural sub-Saharan African population. Despite this level, only 0.5% of children in our study were admitted for malnutrition despite routine weighting of babies. This finding suggests that there must be some difficulties for health care providers in either recognising or referring these children with malnutrition. In our population, weight was the indicator that most clearly showed demonstrable differences between the two groups. Weight is often a more sensitive measure of differences between groups and it is recognised that length is consistently more difficult to measure accurately than weight [23]; this may reflect why significant differences between the groups were seen for WAZ and WLZ but not LAZ. The higher-than-expected levels of low WLZs in our population may also be a reflection of this. It may be that we were observing more acute changes in malnutrition in our sample. We did not have accurate birth weights on enough of the infants to be able to measure change in weight over time between the two groups. Therefore it is not clear from our study whether the significant differences in WAZs between the two groups may be more innately related to preterm infants also being born small for gestational age and never catching up. There were no significant differences in breastfeeding rates at each of the ages assessed to account for the differences in weight between the two groups. We did not have information about supplementary feeding and age of introduction. Considering our findings however, longitudinal studies in this population using both ultrasound to measure gestational age, birth weights, and serial growth measurements with clearer assessments of breastfeeding and complementary feeds could clarify some of these issues.

Infants born preterm were twice as likely to be identified as having a disability using the TQQ as well as to fail the MDAT. Studies from high-income countries have demonstrated educational difficulties in children born late preterm. As far as we are aware, there are no similar studies on developmental outcomes in cohorts of accurately gestationally dated late preterm-born infants from a low-income setting. A study in Bangladesh measured development using a variety of more complex assessment tools adapted from those in high-income countries (Bayley II and Stanford Binet) [12]. This study demonstrated significant differences in development, at a slightly higher level than ours, but the babies were much more preterm (<33 wk gestation) and dated using proxy measures. We have shown that a two-stage approach [42] using the TQQ followed by a more detailed developmental assessment using culturally appropriate assessment tools (such as the MDAT) is feasible even in the most rural settings. Caution is needed, however, when attributing importance to these early assessments of mild impairment as found by using a developmental assessment tool such as the MDAT. Follow-up of this cohort beyond the preschool period would be extremely beneficial to assess the actual amount of impairment, particularly as we found no differences in severe delay between the preterm and the term groups.

It is widely recognised that both nutrition and prematurity have an impact on development. We confirmed that these associations are present in this population from sub-Saharan Africa, but the cross-sectional nature of this study did not allow us to evaluate this relationship in more detail—this would clearly be an interesting area for further research. We suggest that in the absence of more sophisticated screening mechanisms, weight and development could be used as “morbidity markers” and serve as an entry point for identifying other health needs especially for babies born preterm.

### Conclusions

To date, interventions in low-income settings to reduce neonatal morbidity and mortality have targeted the perinatal period. Our data show that, for surviving preterm babies who survive the immediate neonatal period, even in this mainly late preterm-born surviving group, there is ongoing disadvantage with increased risk of death, growth retardation, and developmental delay. Further detailed qualitative and longitudinal studies to assess the causal mechanisms for these problems would be extremely beneficial. Along with these studies, post-neonatal interventions need to be trialled that might improve outcomes in this group of preterm-born children.

## Abbreviations

LAZ: length-for-age z score
MDAT: Malawi Developmental Assessment Tool
OR: odds ratio
SD: standard deviation
TQQ: Ten Question Questionnaire
WAZ: weight-for-age z score
WLZ: weight-for-length z score
